# Leukocyte telomere length and left ventricular function after acute ST-elevation myocardial infarction: data from the glycometabolic intervention as adjunct to primary coronary intervention in ST elevation myocardial infarction (GIPS-III) trial

**DOI:** 10.1007/s00392-015-0848-x

**Published:** 2015-04-04

**Authors:** Vincent G. Haver, Minke H. T. Hartman, Irene Mateo Leach, Erik Lipsic, Chris P. Lexis, Dirk J. van Veldhuisen, Wiek H. van Gilst, Iwan C. van der Horst, Pim van der Harst

**Affiliations:** 1 Department of Cardiology, University Medical Center Groningen, University of Groningen, Hanzeplein 1, 9700 RB Groningen, The Netherlands; 2 Department of Intensive Care, University Medical Center Groningen, University of Groningen, Groningen, The Netherlands; 3 Department of Genetics, University Medical Center Groningen, University of Groningen, Groningen, The Netherlands

**Keywords:** Telomeres, ST-elevation myocardial infarction, Left ventricular ejection fraction, Metformin

## Abstract

**Background:**

Telomere length has been associated with coronary artery disease and heart failure. We studied whether leukocyte telomere length is associated with left ventricular ejection fraction (LVEF) after ST-elevation myocardial infarction (STEMI).

**Methods and results:**

Leukocyte telomere length (LTL) was determined using the monochrome multiplex quantitative PCR method in 353 patients participating in the glycometabolic intervention as adjunct to primary percutaneous coronary intervention in STEMI III trial. LVEF was assessed by magnetic resonance imaging. The mean age of patients was 58.9 ± 11.6 years, 75 % were male. In age- and gender-adjusted models, LTL at baseline was significantly associated with age (beta ± standard error; −0.33 ± 0.01; *P* < 0.01), gender (0.15 ± 0.03; *P* < 0.01), TIMI flow pre-PCI (0.05 ± 0.03; *P* < 0.01), TIMI flow post-PCI (0.03 ± 0.04; *P* < 0.01), myocardial blush grade (−0.05 ± 0.07; *P* < 0.01), serum glucose levels (−0.11 ± 0.01; *P* = 0.03), and total leukocyte count (−0.11 ± 0.01; *P* = 0.04). At 4 months after STEMI, LVEF was well preserved (54.1 ± 8.4 %) and was not associated with baseline LTL (*P* = 0.95). Baseline LTL was associated with n-terminal pro-brain natriuretic peptide (NT-proBNP) at 4 months (−0.14 ± 0.01; *P* = 0.02), albeit not independent for age and gender.

**Conclusion:**

Our study does not support a role for LTL as a causal factor related to left ventricular ejection fraction after STEMI.

## Introduction

ST-segment elevation myocardial infarction (STEMI) is a life-threatening medical condition with a high incidence in Western societies [1]. Timely reperfusion of the culprit artery by primary percutaneous coronary intervention (PCI) is the cornerstone of medical treatment to improve survival and reduce the risk of left ventricular (LV) dysfunction [2]. Nevertheless, up to 30 % of patients develop systolic LV dysfunction after STEMI [3], which is an important predictor for clinical outcome [4]. However, the susceptibility to develop LV dysfunction among individuals suffering from STEMI remains partially unpredictable, even after considering factors as ischemic time and culprit lesion characteristics. Increasing our knowledge on these factors might provide novel avenues for risk stratification and future development of therapy.

We hypothesize that telomere length might be an important factor associated with the development of LV dysfunction after STEMI. In humans, telomeres are repetitive hexameric sequences (TTAGGG)_*n*_ located at the terminal end of chromosomes, which protect genes from degradation during cell division due to the ‘end replication problem’ [5, 6]. With each mitotic cell division, a terminal part of the telomere is lost since DNA polymerases fail to completely replicate the strand which begins at the 3′ chromosomal end [7]. Aging is, therefore, associated with gradual loss of telomere length. If a critical telomere length is reached, cellular senescence or apoptosis is induced [8]. Environmental stressors, for example oxidative stress [9, 10], and inflammatory processes [11], are associated with accelerated shortening of telomere length. Patients with cardiovascular diseases, like coronary artery disease [12], myocardial infarction [13], and heart failure [14] are characterized by shorter telomeres compared to healthy controls [6]. Telomere length has also been associated with LVEF in octogenarians in a non-STEMI setting [15], nevertheless PCI treatment for STEMI has been proven safe and effective in this age group [16, 17]. In addition, genetic variants implicated in LTL have also been associated with LVEF suggesting a potential causal relationship [18].

We present a sub-study of the glycometabolic intervention as adjunct to primary coronary intervention in STEMI (GIPS-III) trial in which we measured leukocyte telomere length to investigate whether baseline leukocyte telomere length is associated with LVEF 4 months after STEMI.

## Methods

### Study population

The design and primary outcomes of the GIPS-III trial have been published previously [19, 20]. In brief, the GIPS-III was a double-blinded, placebo-controlled trial including 380 non-diabetic STEMI patients undergoing PCI and who were subsequently randomly assigned to metformin (*N* = 191) or placebo (*N* = 189) treatment, twice daily for a period of 4 months. Major exclusion criteria included (1) known diabetes, (2) previous myocardial infarction, (3) the need for coronary artery bypass surgery (CABG), (4) severe renal dysfunction, and (5) standard contraindications for magnetic resonance imaging (MRI). The primary outcome was LVEF 4 months after STEMI. After 4 months, LVEF of metformin and placebo-treated patients was similar [20]. All investigators of the GIPS-III trial can be found in the “Appendix.” The trial is registered with clinicaltrials.gov identifier: NCT01217307.

### Study outcomes

Primary study outcome was LVEF determined 4 months after STEMI using a 3.0 T whole-body MRI (Achieva; Philips) using a phased array cardiac receiver coil. Secondary outcomes were other MRI measured parameters [left ventricular end diastolic volume (LVEDV), left ventricular end systolic volume (LVESV), left ventricular end diastolic mass (LVEDM)], and n-terminal pro-brain natriuretic peptide (NT-proBNP).

### Telomere length measurements

Blood for DNA isolation was collected from the patients at arrival at the catheterization laboratory (baseline) which was used for telomere length determination. White blood cell DNA extraction was performed by LGC genomics. Telomere length was measured in quadruplicate on 4 different plates with each replicate in the same well position on the polymerase chain reaction (PCR) plate by the monochrome multiplex quantitative PCR method, originally developed by Cawthon [21]. The telomere primers were TelC: 5′-TGT TAG GTA TCC CTA TCC CTA TCC CTA TCC CTA TCC CTA ACA-3′ (final concentration 900 nM); TelG: 5′-ACA CTA AGG TTT GGG TTT GGG TTT GGG TTT GGG TTA GTG T-3′ (900 nM); the albumin primers were AlbDgc: 5′-GCC CGG CCC GCC GCG CCC GTC CCG CCG GAA AAG CAT GGT CGC CTG TT-3′ (300 nM); AlbUgc: 5′-CGG CGG CGG GCG GCG CGG GCT GGG CGG AAA TGC TGC ACA GAA TCC TTG-3′ (300 nM). The final concentrations of the reagentia per 10 µl reaction were 1X Titanium^®^ *Taq* DNA Polymerase (Clontech Laboratories, Inc.); 1X Titanium^®^ *Taq* PCR Buffer (Clontech Laboratories, Inc.); 0.2 mM of each dNTP (Promega); 0.75X SYBR^®^ Green I nucleic acid gel stain (Sigma-Aldrich); 1 M Betaine (Sigma-Aldrich); 1 mM DL-Dithiothreitol (Sigma-Aldrich). DNA of a human leukemia cell line (1301) with extreme long telomeres was used as a positive control [22]. The thermal cycling profile was done using the BioRad C1000 Touch Thermal Cycler as follows: stage 1: 15 min at 95 °C; stage 2: 2 cycles of 15 s at 94 °C, 15 s at 49 °C; stage 3: 32 cycles of 15 s at 94 °C, 10 s at 60 °C, 15 s at 72 °C with signal acquisition, 10 s at 85 °C, and 15 s at 89 °C with signal acquisition. The T/S ratio was calculated by dividing the telomere (T) signal by the signal of a reference gene (albumin, S). The CFX Manager version 3.0 software was used for generating the standard curves and analyzing the samples. Two standard curves were generated for each plate, one for the telomere signal and one for the albumin signal. Each sample was assayed in triplicate; therefore three T/S ratios were obtained for each sample and the mean of these three T/S ratios was reported. We expect that the mean T/S ratio is proportional to the mean telomere length per cell. If the sample has a T/S ratio >1.0 then the mean telomere length will be longer than the standard DNA; if the sample has a T/S ratio <1.0, the mean telomere length will be shorter than the standard DNA. This T/S ratio, hereafter called leukocyte telomere length (LTL), is a relative measurement of leukocyte telomere content in a sample, which serves as a proxy for actual leukocyte telomere lengths [21]. The median intra-assay coefficients of variation were 9.4 % for T, 10.1 % for S, and 3.4 % for the T/S ratio. Samples were excluded from further analyses if the coefficient of variation for the T/S ratio was >0.1 after deletion of one of the four replicate measurements.

### Statistical analysis

Continuous variables are reported as mean (standard deviation, SD) for normally distributed data. Since LTL and NT-proBNP were non-normally distributed, log transformation was performed to obtain a near normal distribution. Outliers were defined as >2 SD from the median of LTL. For continuous and dichotomous data, we performed linear regression analyses using LTL as dependent variable and baseline characteristics and outcome parameters as independent variables; categorical data were tested using expanded interaction linear regression analyses. All analyses were first performed univariately and then adjusted for age and gender. Graphical representation of interaction analyses were performed using the “margins” command in STATA. Statistical tests were performed two-tailed and a *P* value of <0.05 was used as nominal level of statistical significance. The analyses were performed using StataMP version 13.1 (StataCorp).

## Results

### Study population

Genomic DNA was successfully extracted from 362 (95.5 %) patients of the GIPS-III cohort. LTL was successfully determined in 356 (98.3 %) of the DNA samples (3 samples exhibited insufficient DNA quality, 3 samples were excluded due to coefficient of variation >0.1 after repeated measurement). ANOVA test revealed no significant difference between LTL of both treatment groups (*P* = 0.15). Another 3 samples were regarded as outliers based on >2 SD deviation of the mean LTL, leaving 353 samples for the current analyses. MRI data at 4 months after STEMI were available for 253 (71.6 %) patients of patients whose LTL was determined. Baseline characteristics of the study cohort are represented in Table 1. Patients were aged on average 58.9 ± 11.6 years old and 75.1 % was male. The majority had hypercholesterolemia (62.6 %) and was an active smoker (54.7 %) at baseline. Systolic blood pressure was 134.0 ± 23.5 mmHg and diastolic blood pressure was 84.0 ± 14.4 mmHg. The majority (68.6 %) of the patients presented with single vessel disease. The culprit vessel was predominantly the right coronary artery (RCA). At baseline, NT-proBNP levels were 80 U/L (IQR 38–179), and CK-MB levels were 16 U/L (IQR 13–24).

**Table 1:** Baseline characteristics

Variable	Level	Value
*N*		353
Age (years), mean (SD)		58.9 (11.6)
Gender	Male	265 (75.1 %)
	Female	88 (24.9 %)
Body mass index (kg/m^2^), mean (SD)		26.9 (3.7)
Ethnicity	Caucasian	339 (96.0 %)
	Asian	10 (2.8 %)
	Black	4 (1.1 %)
Hypertension	No	250 (70.8 %)
	Yes	103 (29.2 %)
Hypercholesterolemia	No	132 (37.4 %)
	Yes	221 (62.6 %)
Active smoker	No	160 (45.3 %)
	Yes	193 (54.7 %)
Stroke	No	350 (99.2 %)
	Yes	3 (0.8 %)
Previous PTCA	No	349 (98.9 %)
	Yes	4 (1.1 %)
Systolic blood pressure (mmHg), mean (SD)		134.1 (23.5)
Diastolic blood pressure (mmHg), mean (SD)		84.0 (14.4)
Heart rate (bpm), mean (SD)		75.4 (16.0)
Total ischemic time (min), median (IQR)		161 (109, 251)
Single vessel disease	No	111 (31.4 %)
	Yes	242 (68.6 %)
Culprit vessel	LAD	135 (38.2 %)
	LCX	60 (17.0 %)
	RCA	158 (44.8 %)
TIMI flow grade (pre-interventional)	0	195 (55.2 %)
	1	26 (7.4 %)
	2	60 (17.0 %)
	3	72 (20.4 %)
TIMI flow grade (post-interventional)	2	33 (9.3 %)
	3	320 (90.7 %)
Myocardial blush grade	0	9 (2.6 %)
	1	27 (7.7 %)
	2	70 (20.0 %)
	3	244 (69.7 %)
CK total (U/L), median (IQR)		129 (83, 208)
CK-MB (U/L), median (IQR)		16 (13, 24)
AUC CK total (U × h/L), median (IQR)		1.0 × 10^8^ (4.0 × 10^7^, 2.3 × 10^8^)
AUC CK-MB (U × h/L), median (IQR)		9.8 × 10^6^ (4.2 × 10^6^, 2.0 × 10^7^)
Creatinine (μmol/L), median (IQR)		72 (62, 82)
NT-proBNP (ng/L), median (IQR)		80 (38, 179)
Total leukocyte count (10^−9^/L), median (IQR)		11 (8.8, 13.6)
Glucose (mmol/L), median (IQR)		8.2 (7, 9.5)
HBA1c (%), median (IQR)		5.8 (5.6, 6)

Body mass index was calculated by dividing weight (in kilograms) by squared height (in meters). Normally distributed data are expressed as mean (standard deviation), non-Gaussian data as median (inter-quartile range)

*AUC* area under the curve, *BP* blood pressure, *BMI* body mass index, *eGFR* estimate glomerular filtration rate, *HF* heart failure, *HFrEF* heart failure with reduced ejection fraction, *HFpEF* heart failure with preserved ejection fraction, *hs-CRP* highly sensitive C-reactive protein, *IQR* inter-quartile range, *LAD* left anterior descending coronary artery, *LCX* left circumflex coronary artery, *NT-pro-BNP* N-terminal pro-B-type natriuretic peptide, *RCA* right coronary artery, *SD* standard deviation, *TIMI* thrombolysis in myocardial infarction

### Associations between baseline patient characteristics and LTL

**Fig. 1:**
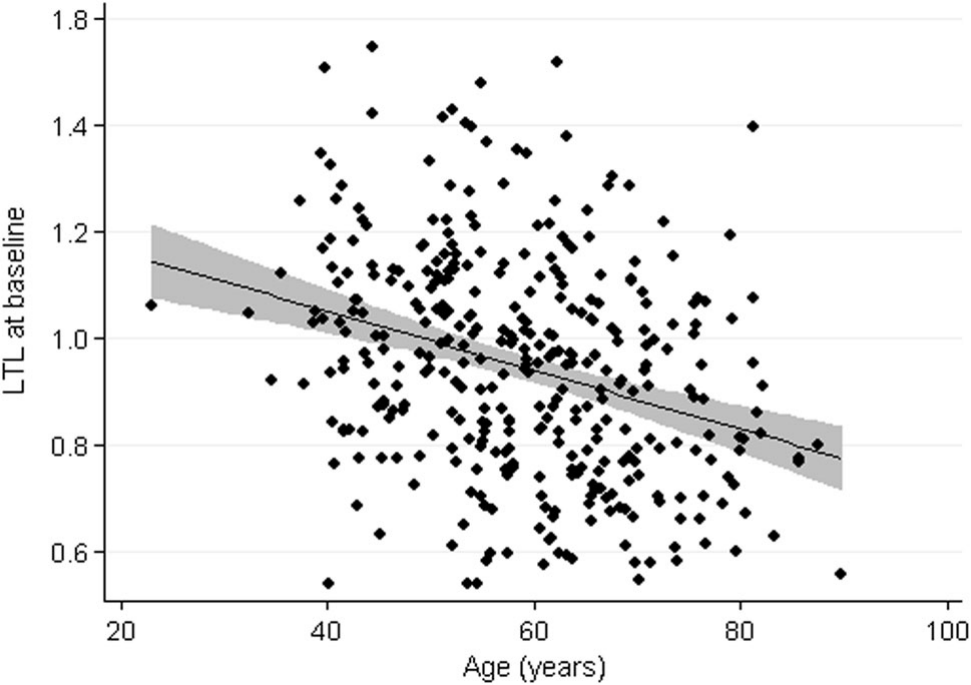
Scatter plot showing association between LTL and age, with superimposed 95 % confidence interval and regression line. *LTL* leukocyte telomere length. Individual data points are shown as well as the superimposed regression line including the 95 % CI

Leukocyte telomere length was negatively associated with age (Fig. 1). Univariate linear regression analyses revealed a significant association between baseline LTL with age, gender, active smoking behavior, single vessel disease, serum creatinine, and glucose levels (Table 2). Although univariately, active smokers seem to have longer LTL than non-smokers, this could be explained by the large age difference between smokers and non-smokers (54.4 ± 10.5 for smokers versus 64.3 ± 10.6 years for non-smokers). After including age and gender in the model, only serum glucose levels remained significantly associated with LTL. Univariately, ‘thrombolysis in myocardial infarction’ (TIMI) flow (both pre- and post-PCI), myocardial blush grade, and total leukocyte count were not associated with baseline LTL; however, after adjustment for age and gender, the association became significant. We tested for an effect of age underlying these association but could not identify a significant interaction effect (interaction coefficient myocardial blush grade = 3.2 × 10^−4^; 95 % confidence interval (CI) −5.5 × 10^−3^ to 6.1 × 10^−3^; *P* = 0.91; interaction coefficient TIMI flow pre-PCI ≤0.01; 95 % CI −0.01 to 0.01; *P* = 0.97; interaction coefficient TIMI flow post-PCI ≤0.01; 95 % CI −0.02 to 0.00; *P* = 0.15; interaction coefficient total leukocyte count = −0.14; 95 % CI −0.29 to 0.01; *P* = 0.08).

**Table 2:** Association of baseline characteristics with LTL

	Univariate model			Multivariate model		
	Std. beta	SE	*P* value	Std. beta	SE	*P* value
Age (years)	−0.31	0.00	<0.01	−0.33	<0.01	<0.01
Gender	0.11	0.03	0.03	0.15	0.03	<0.01
Body mass index (kg/m^2^)	<0.01	<0.01	0.99	−0.04	<0.01	0.42
Ethnicity						
Caucasian			0.18			0.18
Asian	0.01	0.07		0.02	0.07	
Black	0.10	0.11		0.07	0.11	
Hypertension	−0.04	0.03	0.48	0.02	0.03	0.73
Hypercholesterolemia	0.03	0.02	0.58	−0.02	0.02	0.74
Active smoker (y/n)	0.16	0.02	<0.01	0.02	0.03	0.72
Stroke	−0.05	0.13	0.33	−0.02	0.12	0.68
Previous PTCA	−0.02	0.11	0.71	<0.01	0.11	0.99
Systolic blood pressure (mmHg)	−0.02	<0.01	0.77	−0.02	<0.01	0.71
Diastolic blood pressure (mmHg)	0.04	<0.01	0.44	<0.01	<0.01	0.99
Heart rate (bpm)	−0.03	<0.01	0.59	−0.05	<0.01	0.34
Total ischemic time (min)	0.06	<0.01	0.26	0.07	<0.01	0.16
Single vessel disease	0.14	0.03	0.01	0.10	0.02	0.05
Culprit vessel						
LAD			0.51			0.73
CX	0.07	0.03		0.06	0.03	
RCA	0.03	0.03		0.02	0.02	
TIMI flow (pre-PCI)						
0			0.10			<0.01
1	−0.06	0.05		−0.05	0.04	
2	−0.10	0.03		−0.05	0.03	
3	0.05	0.03		0.05	0.03	
TIMI flow (post-PCI)						
2			0.20			<0.01
3	0.07	0.04		0.03	0.04	
Myocardial blush grade						
0			0.82			<0.01
1	0.02	0.09		−0.05	0.08	
2	0.09	0.08		−0.04	0.08	
3	0.11	0.08		−0.05	0.07	
CK total (U/L)	<0.01	<0.01	0.97	0.02	<0.01	0.71
CK-MB (U/L)	−0.01	<0.01	0.92	0.01	<0.01	0.81
AUC CK (U × h/L)	0.03	<0.01	0.58	0.05	<0.01	0.33
AUC CK-MB (U × h/L)	−0.02	<0.01	0.78	0.02	<0.01	0.69
Creatinine (μmol/L)	−0.13	<0.01	0.02	−0.04	<0.01	0.52
NT-proBNP (ng/L)	−0.04	<0.01	0.49	−0.02	<0.01	0.64
Total leukocyte count (10^9^/L)	−0.02	<0.01	0.78	−0.11	<0.01	0.04
Glucose (mmol/L)	−0.15	<0.01	<0.01	−0.11	<0.01	0.03
HBA1c (%)	−0.05	0.01	0.39	−0.02	0.01	0.64

Linear regression analyses of baseline characteristics with LTL are presented for dichotomous and continuous variables; categorical variables were tested by interaction expanded linear regression analyses. Standardized (Std.) beta, standard error (SE), and *P* values are shown. Multivariate tests were adjusted for age and gender (except for age and gender, which were only adjusted for age (gender) or gender (age). Body mass index was calculated by dividing weight (in kilograms) by squared height (in meters)

*AUC* area under the curve, *CK* creatine kinase, *CK-MB* creatine kinase myocardial band, *HBA1c* glycated hemoglobin, *LAD* left anterior descending coronary artery, *LCX* left circumflex coronary artery, *NT-proBNP* n-terminal pro-brain natriuretic peptide, *PTCA* percutaneous transluminal coronary angioplasty, *RCA* right coronary artery, *SE* standard error, *TIMI* thrombolysis in myocardial infarction

### Cardiac MRI at 4 months after STEMI and associations with baseline LTL

**Fig. 2:**
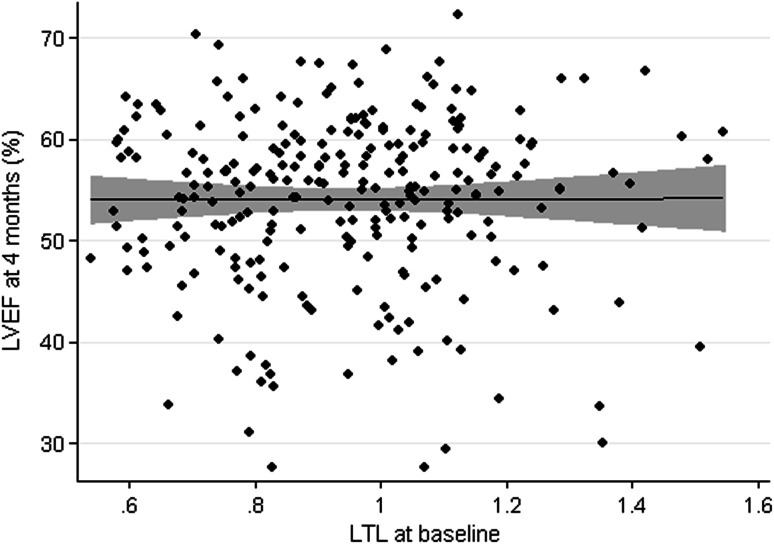
Scatter plot *graph* showing no association between LTL and LVEF at 4 months with superimposed 95 % CI and regression line. *LVEF* left ventricular ejection fraction, *LTL* leukocyte telomere length. Individual data points are shown as well as the superimposed regression line including the 95 % CI

Mean LVEF, as determined by MRI, was well preserved at 4 months after STEMI (54.1 ± 8.4 %). LVEF, left ventricular end diastolic volume (LVEDV), left ventricular end systolic volume (LVESV), left ventricular end diastolic mass (LVEDM), and infarct size are represented in Table 3. LTL measurement at baseline was not associated with LVEF at 4 months (Fig. 2), neither with the other parameters of cardiac remodeling (Table 4).

**Table 3:** STEMI outcomes at 4 months after STEMI

Outcome	Values
LVEF (%)	54.1 (8.4)
LVEDV (mL)	193.4 (45.1)
LVESV (mL)	90.8 (35.5)
LVEDM (g)	100.9 (23.1)
Infarct size (g)	9.3 (9.0)
NT-proBNP (ng/L)	264 (119–631)

Values are presented as mean (SD), except for NT-proBNP, which is presented as median (IQR)

*LVEF* left ventricular ejection fraction, *LVEDV* left ventricular end diastolic volume, *LVESV* left ventricular end systolic volume, *LVEDM* left ventricular end diastolic mass, *NT-proBNP* n-terminal pro-brain natriuretic peptide

**Table 4:** Associations of baseline LTL measurement with STEMI outcomes 4 months after STEMI

	Univariate analyses			Multivariate analyses			Treatment interaction
	Std. beta	SE	*P* value	Std. beta	SE	*P* value	*P* value
LVEF (%)	0.00	<0.01	0.95	−0.01	<0.01	0.88	0.41
LVEDV (mL)	0.09	<0.01	0.17	0.04	<0.01	0.55	0.37
LVESV (mL)	0.06	<0.01	0.37	0.03	<0.01	0.63	0.31
LVEDM (g)	0.08	<0.01	0.20	0.12	<0.01	0.07	0.18
Infarct size (g)	0.02	<0.01	0.77	0.03	<0.01	0.59	0.30
Log NT-proBNP (ng/L)	−0.14	0.01	0.02	−0.07	0.01	0.25	0.04

Univariate and age + gender-adjusted analyses are presented. *P* for interaction represents *P* value of interaction between outcome parameter, LTL and metformin treatment. Standardized (Std.) beta, standard error (SE), and *P* values are shown. Multivariate tests were adjusted for age and gender. *P* for interaction represents the statistical test for outcome modification by metformin or placebo treatment

*LVEF* left ventricular ejection fraction, *LVEDV* left ventricular end diastolic volume, *LVESV* left ventricular end systolic volume, *LVEDM* left ventricular end diastolic mass, *NT-proBNP* n-terminal pro-brain natriuretic peptide

### Treatment effect of metformin on LTL

**Fig. 3:**
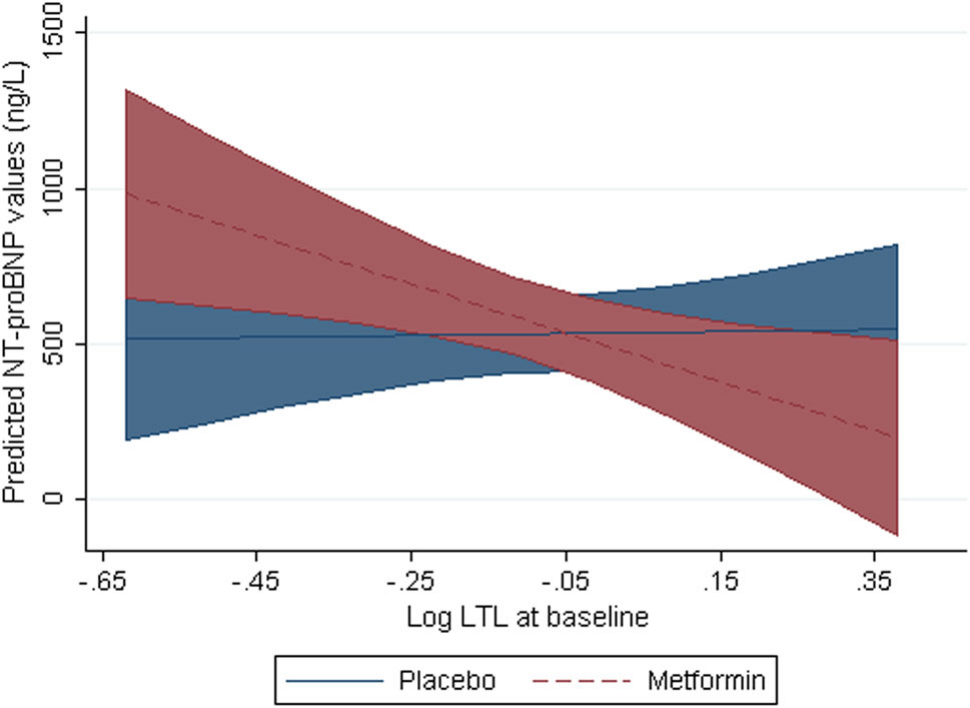
Interaction between baseline LTL and levels of NT-proBNP at 4 months after Metformin or Placebo treatment. *LTL* leukocyte telomere length. Linear prediction represents the predicted NT-proBNP for both metformin as well as placebo-treated patients. Regression line and 95 % CI are shown

We have explored the possible interaction of metformin with LTL on LVEF at 4 months. Interaction analyses revealed no significant interaction of treatment with the association of baseline LTL and LVEF at 4 months (interaction coefficient = 4.0; 95 % CI −5.5 to 13.5; *P* = 0.41, Table 4). However, we found evidence for effect modulation by metformin treatment on the association of LTL with NT-proBNP at 4 months (interaction coefficient = −1.3; 95 % CI −2.5 to −0.1; *P* = 0.04). NT-proBNP levels were similar for patients with different levels of LTL after placebo treatment but in patients treated with metformin, longer LTL was associated with lower NT-proBNP levels (Fig. 3).

## Discussion

Leukocyte telomere length has been proposed as a marker of biological age and has been suggested to play an important role in cellular senescence or apoptosis [8]. Previously, associations have been reported between LTL with coronary artery disease [12], heart failure [23], and LVEF [15]. We hypothesized that LTL is associated with cardiac remodeling after STEMI as can be reflected by LVEF at 4 months. The main finding of the present study is that we could not find support for this hypothesis.

In our study, we did observe the well-established association of LTL with baseline characteristics such as the inverse association with age [23, 24] and gender (females having longer LTL [25]). The direction of smokers was opposite as frequently reported (active smokers in GIPS-III were found to have longer LTL) [22, 26] but this could be completely explained by the large age difference between non-smokers and smokers. These associations suggest that our main finding is unlikely due to measurement error of LTL. A possible explanation for the absence of an association between LTL and LVEF in the GIPS-III trial might be the relatively well-preserved LVEF after STEMI. Considering the mean LVEF of approximately 54 % after STEMI, the variation of the primary endpoint might have been too small to establish an association with LTL. However, even in the absence of STEMI and the resulting cardiac remodeling, one could speculate on an association between LVEF and LTL. In a cohort of octogenarians (*N* = 64; average age 85.2-year old) without evidence of previous myocardial infarction, LTL was strongly and independently associated with LVEF as determined by echocardiography [15]. In this cohort, approximately 12 % of the observed variability in LVEF could be explained by LTL alone. In addition, an association between LTL and LVEF has been reported in subjects with hypertension (*N* = 1106; average age 57.9-year old). A 1.5 fold larger LTL was associated with 0.6 % increase in absolute LVEF [18]. On the other hand, there are also several studies reporting a lack of an association between LTL and LVEF in other settings. In a cohort with established heart failure patients (*N* = 610; average age 66.2-year old), we did not observe an association with LVEF [14]. In another cohort of patients with idiopathic cardiomyopathies (*N* = 223; average age 51.1-year old), LTL was also not associated with LVEF as determined [27]. Also in subjects derived from the general population, the absence of an association between LTL and LVEF has been reported. In the Malmö Preventive Project, a cross-sectional observational study including 1588 subjects (average age 67.7-year old), an association with LTL with LVEF was lacking [28]. In an additional population-based cohort of Chinese Han people (*N* = 139; average age 60.3-year old), there was also no association with LTL and LVEF [29].

Our data contribute to the previous studies by investigating a specific population (STEMI) in which the role of LTL might be more prominent. However, our data demonstrate that even in the setting of STEMI and the subsequent remodeling process of the heart, LTL does not seem to be associated and, therefore, is unlikely to be involved. Biomarkers for predicting outcomes in coronary heart disease outcomes have been reported [30], but the present study does not support the use of LTL as a biomarker in the setting of STEMI. The well-preserved LVEF 4 months after STEMI, which is the result of the high level of acute care in our STEMI network [31], could have nullified the potential role of LTL in STEMI outcome prediction. Besides LVEF, we also took into account possible relationships between LTL and other outcome parameters. In our study, lower levels of NT proBNP, often elevated in heart failure, were associated with longer LTL only in the metformin group. Further studies are needed to clarify if this association can be attributed to the effect of metformin. Other studies report the predictive value of cardiopulmonary exercise testing in heart failure patients with previous myocardial infarction [32]. In experimental mice model, physical activity prevented senescence of leukocytes and increased telomerase activity was seen in endurance athletes [33]. In this study, physical activity was not measured; therefore, we cannot assess the effect of physical activity on LTL. Worsening renal function and acute kidney injury are associated with increased mortality in STEMI patients [34]. In our study, serum creatinine was associated with shorter LTL; however, after adjusted for age and sex, this relationship did not remain significant.

The major limitation of our study that needs to be considered is that the cells we investigated are leukocytes. Therefore, we cannot exclude an important role of telomere length in other cell types, e.g., cardiomyocytes or endothelial cells [35]. For practical (and ethical) reasons, it is not feasible to study cardiomyocytes of STEMI patients. Another limitation is that our analyses are based on a single LTL measurement. Therefore, we cannot exclude that LTL measurements in the stable setting or cross-sectionally at time of LVEF determination are associated with LVEF. This remains to be determined. In this study, PCR method was used for feasibility reasons; however, the gold standard for LTL assessment is FACS-FlowFISH which more specifically can differentiate between different cell subpopulations within the peripheral blood [36]. Finally, telomere length is only one of the parameters of telomere biology related to apoptosis and senescence. Telomere biology is more complex than telomere length alone. It also involves many regulatory and stabilizing protein complexes (sheltering) interacting with the telomere DNA sequence to protect the DNA [6, 37]. The exclusion of telomere length as a factor associated with LVEF does not exclude a role of telomere biology per se. The strengths of our study include that we have executed the current study within the framework of a clinical trial using the golden standard to determine LVEF.

In conclusion, LTL measured in the setting of STEMI is not associated with cardiac remodeling or LVEF as determined by MRI after 4 months. Our study does not lend support for a role of LTL as a causal factor in LV remodeling or for the use as a biomarker to predict clinical outcome in patients with acute myocardial infarction.

## Appendix

Members of the GIPS-III Investigator group are as follows: Publication/Writing Committee: I. C. C. van der Horst (chair), C. P. H. Lexis, D. J. van Veldhuisen, E. Lipsic, P. van der Harst, H. L. Hillege, J. G. P. Tijssen; Steering Committee: I. C. C. van der Horst (chair), D. J. van Veldhuisen, E. Lipsic, P. van der Harst, R. A. de Boer, A. N. A. van der Horst-Schrivers, B. H. R. Wolffenbuttel; Adjudication Committee: F. van den Berg, V. M. Roolvink, A. P. van Beek; Data Safety Monitoring Board: J. G. P Tijssen (chair), R. J. de Winter, A. J. Risselada, R. M. de Jong, R. K. Gonera; Investigators: all in the Netherlands: *University Medical Center Groningen, Groningen—*I. C. C. van Horst, C. P. H. Lexis, E. Lipsic, P. van der Harst, D. J. van Veldhuisen, A. F. M. van den Heuvel, W. G. Wieringa, H. W. van der Werf, Y. Tan, G. P. Pundziute, R. A. J. Schurer, (B. J. G. L. de Smet), A. N. A. van der Horst-Schrivers, B. H. R. Wolffenbuttel, W. Nieuwland, P. van der Meer, R. A. Tio, J. Coster, (A. A. Voors, J. P. van Melle, Y. M. Hummel) B. H. W. Molmans, *University of Groningen, Groningen*—G. J. ter Horst, R. Renken, A. J. Sibeijn-Kuiper; *VU University Medical Center, Amsterdam*—A. C. van Rossum, R. Nijveldt; *Academic Medical Center, Amsterdam*—J. G. P Tijssen.
